# Imaging Point Source Groundwater Discharges in a Confined Coastal Aquifer Using Electrical Resistivity

**DOI:** 10.1111/gwat.70071

**Published:** 2026-04-27

**Authors:** Mariana Gómez‐Nicolás, Armando Huitzilt‐Rodriguez, Enrique Iñiguez, Roger Pacheco‐Castro, Marco Pérez‐Flores, Paulo Salles, Fiona Whitaker

**Affiliations:** ^1^ CICESE División de Ciencias de la Tierra, km 107 Carretera Tijuana‐Ensenada Baja California 22680 México; ^2^ Facultad de Ciencias Universidad Nacional Autónoma de México (UNAM) Coyoacán 04510 Ciudad de México México; ^3^ SECIHTI—Center for Scientific Research and Higher Education Geology Department, Carretera Ensenada Tijuana #3918 Zona Playitas CP: 22860 Baja California México; ^4^ Currently at Centro de Investigaciones Químicas Universidad Autónoma del Estado de Morelos Cuernavaca Morelos México; ^5^ Laboratorio de Ingeniería y Procesos Costeros, Instituto de Ingeniería Universidad Nacional Autónoma de México Puerto de Abrigo S/N Sisal 97355 México; ^6^ Laboratorio Nacional de Resiliencia Costera Sisal 97355 Mexico; ^7^ Investigadoras e Investigadores por México, SECIHTI Ciudad de México México

## Abstract

Point‐source submarine groundwater discharge (PSGD) delivers mass and solutes and locally reduces salinity along karstic coasts, yet the geometry of conduits conveying meteoric and mixed waters is rarely imaged. Here we present the first marine electrical resistivity tomography (ERT) characterization of PSGD along the northwestern Yucatán Peninsula and evaluate marine ERT across contrasting settings to detect and characterize PSGD conduits and the confining coastal aquitard under field conditions. Four dipole–dipole ERT profiles span an intact coastal aquitard on land (Sisal), a coastal lagoon perforated by PSGD (Dzul‐Ha), and their offshore equivalents near the Xbuya‐Ha vent. On land, a laterally continuous resistive horizon at −5 to −9 masl overlies low‐resistivity units that host saline groundwater and is interpreted as a cemented coastal aquitard that confines the aquifer. Beneath Dzul‐Ha, the inversion images an elongated conductive chimney that cross‐cuts this horizon and coincides with the mapped PSGD. Seabed profiles near Xbuya‐Ha resolve discrete resistive bodies embedded in a conductive matrix at the main and secondary PSGDs. Forward modeling shows that conduits remain detectable even when the salinity contrast generated by freshwater discharge is weak; however, anomaly amplitudes decrease with increasing sea‐floor depth, following an exponential decay with a characteristic depth of ~8 m. Together, these results provide a resistivity‐based framework for combining tracers and flow models to quantify PSGD fluxes. Because the study targets vigorous PSGD under favorable electrical contrasts, these performance estimates represent an upper bound and clarify the limits of marine ERT for detecting PSGD in comparable karstic coasts.

## Graphical abstract

Marine electrical resistivity tomography (ERT) is applied to characterize point‐source submarine groundwater discharge (PSGD) along NW Yucatán. ERT observations and forward modeling constrain conduit detectability under varying hydrogeological conditions. Results define practical detection limits, highlight depth‐dependent signal attenuation, and support interpretation of PSGD in coastal karst aquifers.

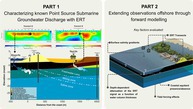

## Introduction

Offshore freshened groundwater (OFG)—part of the broader category of “Vast Meteoric Groundwater Reserves” that contain water with <10 g L^−1^ total‐dissolved solids—is now recognized beneath many continental shelves worldwide (Post et al. 2013; Knight et al. 2018). Discharge of OFG represents only 0.01‐10% of river runoff globally but, in combination with recirculated seawater, the total submarine groundwater discharge (SGD) can equal or exceed river discharge (Jiao and Post 2019). SGD is now recognized as a major pathway for dissolved solutes and nutrients to the coastal ocean, often rivaling or exceeding riverine inputs (Santos et al. 2021). A recent global synthesis indicates that SGD‐derived nutrient fluxes exceeded riverine inputs in ~60% of the coastal sites examined, underscoring the importance of this pathway even where surface runoff is present (Santos et al. 2021). In carbonate terranes that characteristically lack surface rivers, SGD constitutes the sole pathway for mass and solute transfer to the oceans, making its role in coastal biogeochemistry particularly significant. Although not all SGD is enriched in nutrients or contaminants, discharges that are nutrient and pollutant rich can substantially influence coastal productivity, nutrient ratios, and benthic ecology in ways that are often underestimated (Jiao and Post 2019).

SGD occurs in a wide range of geological settings, including permeable sandy shelves and rocky or karstic coasts that do not necessarily include a coastal aquitard (Whitman and Yeboah‐Forson 2015; Santos et al. 2021). In this study we focus specifically on the offshore extension of low‐salinity groundwater where the presence of a coastal aquitard with low vertical hydraulic conductivity can be a primary control on the offshore extent and preservation of upward freshwater discharge (Marksamer et al. 2007; Cohen et al. 2010; Santos et al. 2021). Such aquitards may be of depositional origin—for example, offshore New Zealand (Micallef et al. 2021)—or diagenetic, where cementation produces laterally extensive hardgrounds, as in Abu Dhabi (Vallack 2021). Numerous studies, including those along the coasts of Hong Kong, Suriname, and the Atlantic continental shelf of the USA, illustrate how laterally extensive low‐permeability layers inhibit mixing between offshore fresh groundwater and overlying seawater (Solórzano‐Rivas and Werner 2018). When pressure gradients or buoyancy drive this confined OFG toward the seabed, the permeability distribution determines the fraction that emerges as focused springs and as diffuse seepage (Fu et al. 2020; Zhang et al. 2024). Along the north‐western coast of the Yucatán Peninsula, a diagenetic coastal aquitard has been documented (Perry et al. 1989), and its low vertical hydraulic conductivity may govern the offshore extent and magnitude of freshwater discharge. Karstic dissolution locally perforates this aquitard, focusing confined flow into PSGD that act as preferential‐flow pathways and influence nearshore water balance and ecosystem health (Finch 1965; Pacheco‐Castro et al. 2021).

Geophysical imaging has become indispensable for locating OFG bodies and their discharge pathways. Electrical‐resistivity imaging can detect variations in subsurface conductivity produced by changes in pore‐water salinity and lithological contrast (Singha et al. 2022). Marine adaptations of electrical resistivity, including continuous resistivity profiling and towed transient electromagnetics, offer a cost‐effective way to delineate sharp salinity contrasts (Gustafson et al. 2019; Micallef et al. 2021). Electrical‐resistivity imaging has been used to map groundwater/seawater interfaces, monitor dynamic SGD processes, and characterize complex multilayer aquifer–aquitard systems (O'Connell et al. 2018; Fu et al. 2020; Zhan et al. 2022). Such resistivity images can resolve confined aquifers, low‐permeability aquitards, and fracture networks that modulate saline intrusion or focus PSGD (Ritzi et al. 2001; Taniguchi et al. 2019). When combined with natural tracers (e.g., radon, radium), the images provide the structural framework needed to refine discharge estimates (Cantarero et al. 2019).

Marine ERT is one of the few hydrogeophysical tools that combines high sensitivity to seawater–freshwater contrasts with commercially available instrumentation and relatively low budget field logistics. Synthetic modeling studies by Loke and Lane (2004), Kwon et al. (2005), Day‐Lewis et al. (2006) and Papadopoulos et al. (2022), established practical array geometries and quantified resolution limits under varying water‐column conductivities and depths. These guidelines underpin an expanding portfolio of SGD studies worldwide reviewed by Taniguchi et al. (2019). However, the inherent smoothness of marine ERT inversions tends to underestimate absolute resistivities in seawater‐saturated units and blur the freshwater–saltwater mixing zone. As a result, resistivity sections are best interpreted together with heads, *in situ* conductivity measurements, and other hydrogeological constraints (Costall et al. 2020). Yet, despite the increasing application of these methods, and the extensive karstification (Bauer‐Gottwein et al. 2011) and importance of SGD along Mexican coasts (Murgulet et al. 2020), no peer‐reviewed marine ERT investigation has been previously reported.

Here we present the first marine ERT characterization of PSGD along the north‐western Yucatán coast, a setting where SGD represents virtually the entire land‐to‐sea solute flux because of the absence of rivers. Our aim is to evaluate how effectively marine ERT can detect, resolve, and interpret PSGD in an area where strong confinement is imposed by the coastal aquitard (Perry et al. 1989), and to determine the limits of the method under realistic field conditions. Specifically, we examine how the electrical response evolves along a natural gradient that spans an intact coastal aquitard on land, zones where this aquitard is breached by dissolution conduits within the coastal lagoon, and finally offshore locations that feature submarine springs. This configuration allows us to assess the sensitivity of marine ERT to the presence of narrow vertical pathways and explore whether a fracture‐guided conduit can produce a resolvable electrical signature. Additionally, we can determine how variations in the salinity structure of the water column, such as generated by active springs, enhance or obscure anomaly detection. Because the regional aquitard has been inferred to extend several kilometers offshore, we also investigate the conditions under which its presence or absence can be distinguished from resistivity data, including the role of sea‐floor depth in attenuating the signal.

By integrating field data with forward modeling, our study establishes a resistivity‐based framework for future tracer, modeling, and multidisciplinary studies aimed at quantifying SGD fluxes in karstic coastal aquifer systems, and to clarify the practical limits of marine ERT for detecting PSGD in such environments. In establishing these constraints, our study provides a valuable reference for future investigations, enabling more informed design and application of marine ERT in coastal aquifer research. Beyond addressing a critical regional knowledge gap with financially accessible technology, our work offers practical guidance for multidisciplinary efforts aimed at quantifying SGD fluxes and managing groundwater resources under increasing anthropogenic pressure.

## Study Zone

**Figure 1:**
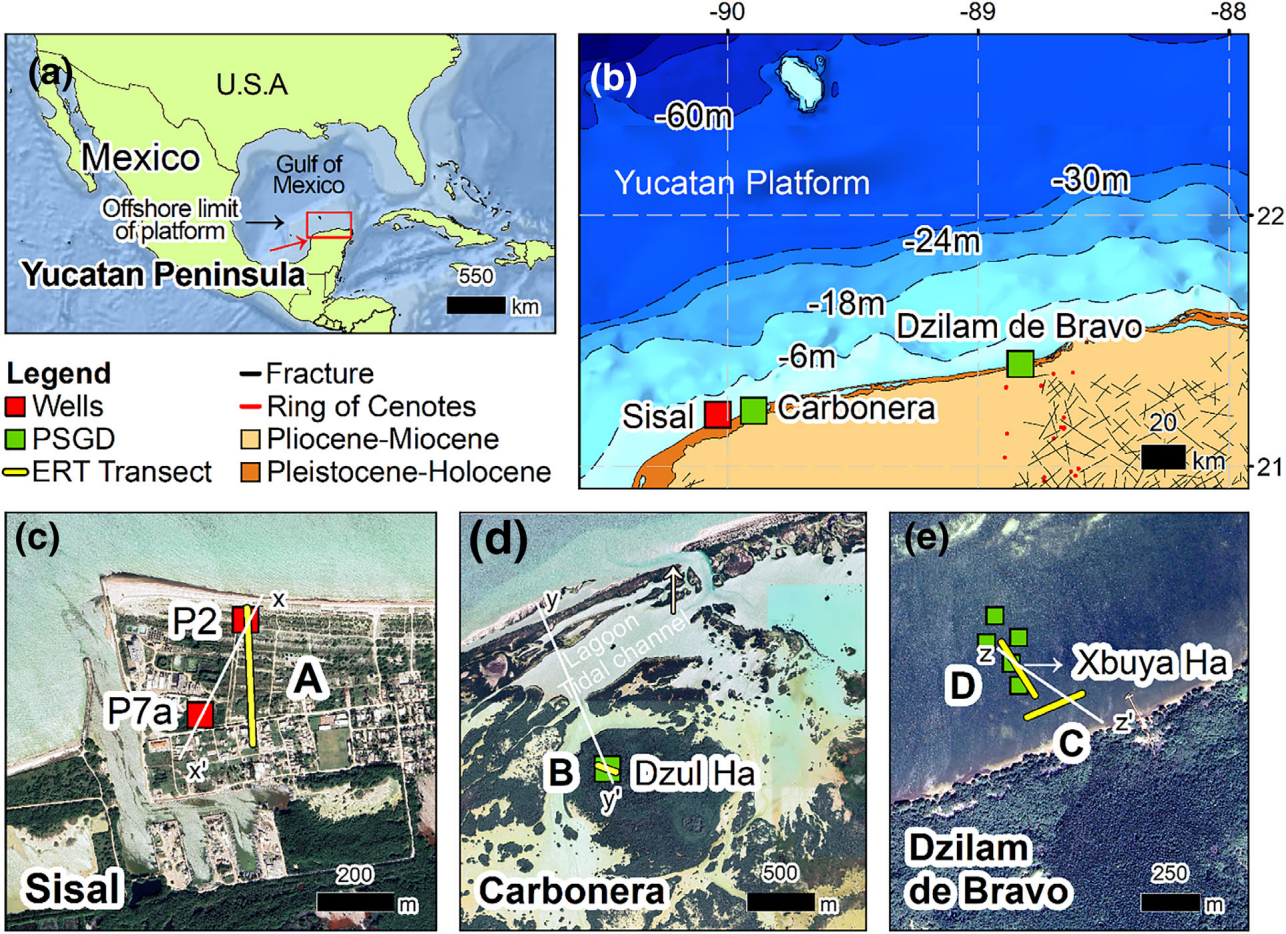
(a) Location of the study area along the northwestern Yucatán Peninsula and the offshore extent of the Yucatán Platform. (b) Regional bathymetry of the Yucatán Platform showing depth contours (−6 to −60 m) and the coastal sector between Sisal, Carbonera, and Dzilam de Bravo. (c‐e) Locations of onshore and marine ERT transects (yellow) at Sisal, Carbonera, and Dzilam de Bravo, superimposed on satellite imagery. Lettered markers (x–x′, y–y′, z–z′) denote the projected positions presented in Figure 2 along the conceptual cross section. Surficial geology is modified from Servicio Geológico Mexicano (2005, 2006a, 2006b) and CONANP (2025); Bathymetry is derived from Shuttle Radar Topography Mission data (NASA 2000); Satellite imagery is from Google Earth (2025).

The Yucatán Peninsula forms the 150,000 km^2^ emergent portion of the Yucatán Platform, separating the Gulf of Mexico from the Caribbean Sea (Figure 1a). A thick sequence of shallow‐water limestones extends to depths of at least 150 m more than 50 km inland of the Caribbean coast, and ~1000 m beneath Mérida in the northwest (Ward 1996). These carbonates host one of the world's largest karstic aquifer systems (Gondwe et al. 2010), In this context, within a highly heterogeneous eogenetic aquifer (Budd and Vacher 1991), conduits and numerous pit cenotes (vertical shafts) intersect the water table and provide access to a vertically stacked network of subhorizontal cave passages developed by mixing zone dissolution (Smart et al. 2006, 1990). A striking arcuate belt of closely spaced pit cenotes, known as the “Ring of Cenotes”, overlies the Chicxulub impact structure (Perry et al. 1995; Pope et al. 1996), enhancing vertical permeability, and likely focusing both onshore spring discharge and offshore SGD (Monroy‐Ríos 2020). Consistent with this hydrogeologic framework, SGD along the coast of the Yucatán Peninsula is thought to be dominated by PSGD, which has been estimated to account for 78‐99% of total SGD (Parra et al. 2015). Within the broad range of coastal aquifers observed globally, the Yucatán Peninsula thus provides a type exemplar of offshore freshened‐groundwater discharge from a highly complex karstified aquifer system.

The study area is located along the northwestern coast of the Yucatán Peninsula between the towns of Sisal, La Carbonera, and Dzilam de Bravo (Figure 1). ERT data were collected along four coastal transects: Transect A in Sisal, Transect B at the Dzul‐Ha petén (mangrove ecosystem forming tree islands sustained by focused groundwater discharge) in La Carbonera; and Transects C and D in Dzilam de Bravo, with Transect D intersecting Xbuya‐Ha spring. The study domain extends from approximately 21°10′ N, 90°03′ W to 21°24′ N, 88°50′ W, encompassing a representative portion of the northern Yucatán carbonate platform.

### Lithostratigraphy, Hydrostratigraphy, Hydraulic Characteristics and Flow Regime

**Figure 2:**
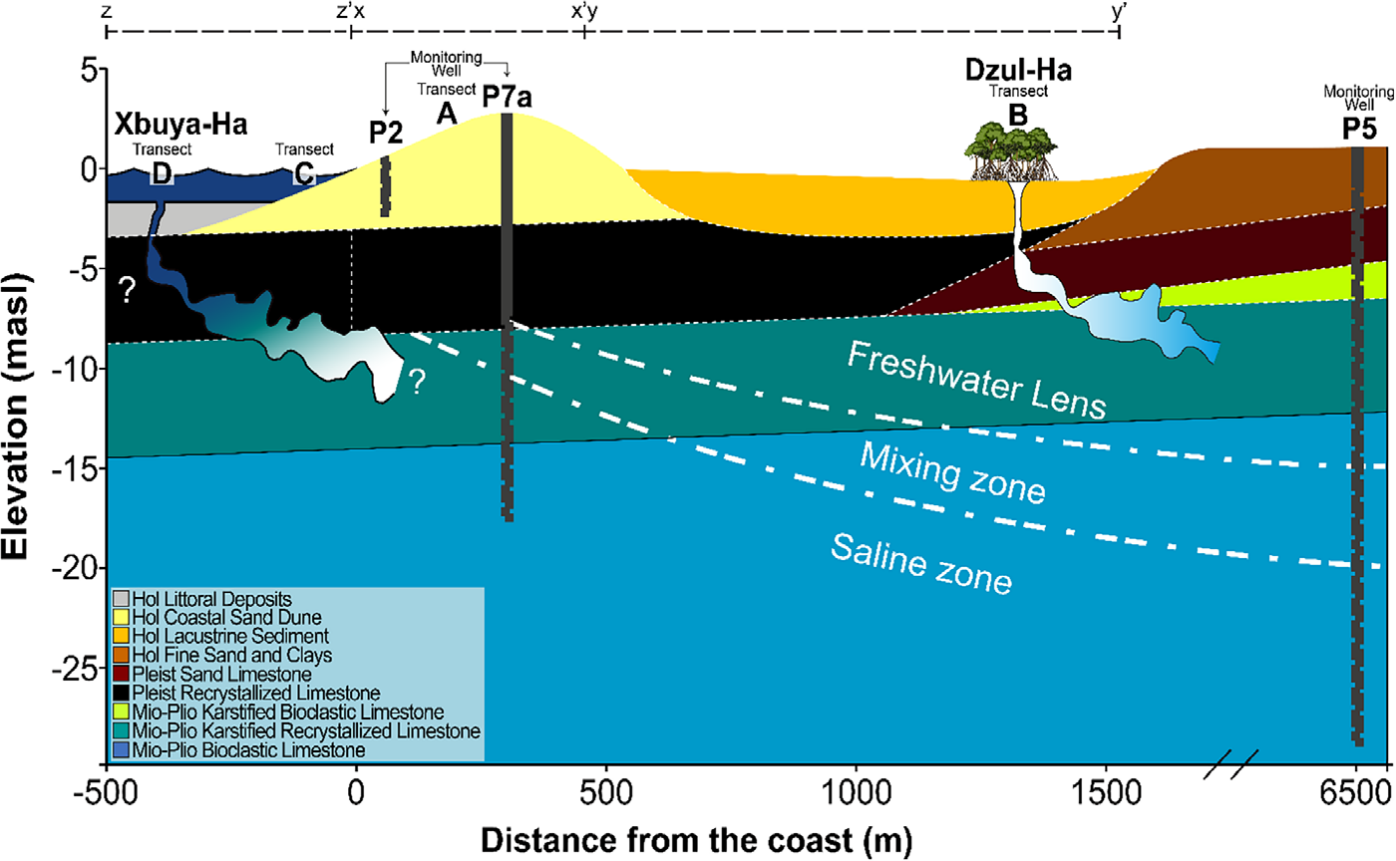
Conceptual cross‐section illustrates the relative positions of study sites across the coastal zone. The diagram integrates monitoring well data and stratigraphic interpretations from Canul‐Macario (2020). Interface lines between these zones are drawn only up to monitoring well P7a and P5, where their positions are constrained by field measurements. PSGD conduits through the coastal aquitard (Pleistocene Recrystallized Limestone, Holocene Fine Sand and clays and Pleistocene Sand Limestone) are represented to indicate inferred pathways for focused groundwater discharge. Modified from Perry et al. (2003) and Canul‐Macario et al. (2020).

The lithostratigraphic and hydrostratigraphic framework adopted in this study (Figure 2) is anchored to the Sisal site (Figure 1c), where data from monitoring wells P7a and P5 (Figure 2) provide the most complete geological control (Canul‐Macario et al. 2020). Regional geological maps and previous stratigraphic studies indicate this roughly horizontally bedded sequence of limestones is broadly consistent along the northern Yucatán coastal zone (Servicio Geológico Mexicano 2005, 2006a, 2006b). At shallow depth this sequence typically comprises recent unlithified deposits that transition from littoral to beach dunes and lacustrine sediments, which overlie a Holocene sandy limestone horizon that acts as a coastal aquitard. This is underlain in turn by recrystallized Miocene–Pliocene fossil‐rich limestones of the Carrillo Puerto Formation (CPF). This is a highly permeable carbonate unit characterized by extensive secondary porosity produced by fracturing, cavities, and dissolution conduits. Owing to its broad regional extent and the shallow position of the water table within it, the CPF constitutes the principal hydrogeological unit of the Yucatán Peninsula (Perera‐Burgos et al. 2024). Across the northern Yucatán, highly porous carbonate facies analogous to this underlying unit, including unconsolidated sascab (friable limestone, Glumac et al. 2018) and fossil‐rich coquina, exhibit matrix porosities on the order of 25‐50% (González‐Herrera et al. 2002; May‐Crespo et al. 2010, 2012; Estrada‐Medina et al. 2013). These units collectively define the principal hydrostratigraphic framework of the northern coastal fringe.

At monitoring well P7a, located ~300 m inland from the shoreline, this sequence is expressed as follows. From 2.9 to approximately −5 m above sea level (masl), the section consists of beige, sandy limestone rich in mollusk and shell fragments, corresponding to Holocene coastal dune deposits. From approximately −5 to −9 masl, a dense, recrystallized carbonate horizon—locally referred to as caliche is encountered below which Canul‐Macario et al. (2020) describes the CPF as comprising a bioclastic limestone, with notable recrystallization at −9‐15 masl, and karstification extending some 6 m below the base of the cemented unit. This layer, interpreted as late Pleistocene in age based on radiocarbon evidence (~23 ky), is thought to have formed via a combination of vadose diagenesis and coastal cementation (Perry et al. 1989). However, its genesis remains under debate (Smart et al. 1990). In this study, we refer to this cemented layer as the coastal aquitard to emphasize its hydrogeological function. This unit exhibits very low porosity (<1%) and hydraulic conductivity (<10^−7^ m s^−1^) based on laboratory‐scale measurements of a ~ 10 cm^3^ rock sample (Canul‐Macario 2020), effectively confining the underlying aquifer in the nearshore zone. Transect A was intentionally positioned ~100 m east of monitoring well P7a and ~5 m from P2, over the same coastal dune deposits, so that its geoelectrical signature could be directly constrained by the well stratigraphy. No fractures or discharge features are known in this sector, and the coastal aquitard is therefore considered intact at this site.

At monitoring well P5, located ~6.4 km inland from the shoreline (ground elevation 0.54 masl) and characterized by a ~0.5 m vadose‐zone thickness, the stratigraphic sequence is expressed as follows. The upper ~2 m of the section consists of a gray sandy–clayey limestone with local recrystallization. Beneath this (~−1.5 to −3.5 masl) a layer of fine‐grained limestone is encountered. This is interpreted here as a zone of recent cementation, where precipitation of pore occluding cements is likely driven by evaporation and degassing of dissolved CO_2_ at and close to the water table (Perry et al. 1989; Smart et al. 1990). This confining horizon is not widespread across the Yucatán Peninsula but is restricted to the northern coastal fringe, extending ~5‐7 km inland (Perry et al. 1989). Although its offshore extent remains poorly constrained, available evidence suggests that it continues an undetermined distance into the Gulf of Mexico (Perry et al. 1989, 2003). From approximately −3.5 to −6.5 masl, the section transitions to a massive cream‐to‐white limestone with coquinoid horizons containing abundant fossils and dissolution features. From approximately −6.5 to −15.5 masl, a compact, recrystallized Miocene–Pliocene limestone of massive appearance is present and is pervasively fractured, corresponding to the CPF. Finally, from approximately −15.5 to −30 masl, a white‐to‐beige, micritic Miocene–Pliocene coquina dominates the lower part of the profile.

Hydrogeologically, two distinct aquifers are present: (1) a shallow, local unconfined aquifer within the Holocene dune deposits, and (2) a deeper, regional karst aquifer hosted in Miocene–Pliocene limestones. These are hydraulically separated in the coastal zone by the coastal aquitard. Regional groundwater flow is oriented from the southeast toward the northwest (with locally coastward and radial flow near the shoreline). Near the coast, a freshwater lens overlies a saline body, with a mixing zone that defines the coastal saline interface (Canul‐Macario et al. 2020).

Hydraulic conductivity shows marked scale dependence: slug‐test estimates in monitoring well P2 are on the order of 10^−5^ m s^−1^ (Medina‐Rosado 2020), while in well p7a values increase to ~10^−3^ to 10^−2^ m s^−1^ depending on the test scale, from slug‐test to pumping tests (Canul‐Macario et al. 2020). The regional aquifer behaves as a confined coastal system that interacts with seawater and shows a gentle continental hydraulic gradient toward the coast (∼0.03 m km^−1^).

### Point Source SGD

Point‐source groundwater discharges in the coastal zone were evaluated both within a coastal lagoon at La Carbonera (Dzul‐Ha PSGD, ERT Transect B) and offshore at Dzilam de Bravo (Xbuya‐Ha PSGD, ERT Transects C and D).

Dzul‐Ha PSGD nestled within the La Carbonera coastal lagoon (Figure 1d) forms an approximately circular pool ~9 m diameter and ~2 m deep (Pacheco‐Castro et al. 2021). It behaves like a classic estavelle; discharging fresh groundwater for much of the time but acting as a sink when lagoon stage rises and flow reverses. Flow at this tidal spring reflects the combined influence of sea‐level oscillations, local recharge/discharge, and partial aquifer confinement (Febles and Batllori 1995; Rey 2012; Pacheco‐Castro et al. 2021). Continuous monitoring shows a clear tidal periodicity superimposed on longer‐term recharge trends, with the resistivity of vent water changing by more than an order of magnitude depending on flow direction. The spring discharges water with a minimum specific electrical conductivity of 3.1 mS cm^−1^ (∼1.6 psu; 3.2 Ω·m), whereas during 3‐7 h reverse‐flow events conductivity increases to a maximum of 37.9 mS cm^−1^ (∼23.5 psu; 0.26 Ω·m) (Pacheco‐Castro et al. 2021). These sharp, tidally driven switches highlight the vulnerability of lagoonal PSGD points to saline intrusion and underline the need to distinguish inland lagoon springs such as Dzul‐Ha from offshore point‐source discharges when estimating regional SGD fluxes.

Xbuya‐Ha—“the water labyrinth” in Mayan—is an offshore PSGD within the municipality of Dzilam de Bravo (Figure 1e), at the eastern intersection of the Ring of Sinkholes with the coast. The spring lies ~350 m offshore in water depths of ~1 m, and consists of a ~ 1 m^2^ opening in the limestone bedrock, with four additional springs occurring within <200 m of the main vent (Valle‐Levinson et al. 2011). Discharge at Xbuya‐Ha is inversely related to ocean tide: low tide produces the strongest outflow, whereas at high tide the flux weakens or even reverses (Valle‐Levinson et al. 2011; Vera et al. 2012). Reported discharge magnitudes range from order 10^4^ m^3^ d^−1^ (<43,200 m^3^ d^−1^; Murgulet et al. 2020) to instantaneous estimates of ~1 m^3^ s^−1^ (Smart et al. 1990), underscoring strong temporal and methodological variability. Given these fluxes, coastal flow and salinity fields are measurably altered by the plume of brackish groundwater. Fresh/brackish outflow can lower the salinity of seawater local to the vent to ~23 psu (∼37.1 mS cm^−1^; 0.27 Ω·m) compared with ambient values of ~36 psu (∼55.5 mS cm^−1^; 0.18 Ω·m). The lowest radium ages and highest SGD rates in the area cluster around this “underwater labyrinth” (Murgulet et al. 2020), underlining rapid flow through the karstified aquifer and short groundwater residence times.

## Methods

ERT transects were acquired by injecting current through a transmitting electrode pair and recording the resulting voltage differences at multiple receiving electrodes arranged in successive four‐electrode configurations (Binley and Slater 2020; Singha et al. 2022). These measurements form the basis for inversion, in which a forward solver computes synthetic apparent resistivities for an initial subsurface model and an iterative inverse algorithm updates that model to reduce the misfit between measured and synthetic responses (Binley and Slater 2020; Singha et al. 2022). During inversion, data uncertainty is accounted for through the weighting parameters *a_wgt* and *b_wgt*. The former represents a constant baseline noise associated with instrumental limitations, whereas the latter captures errors that scale with the magnitude of the measured resistance. Together, these parameters define the standard deviation assigned to each data point and therefore control its relative influence on the inversion, ensuring that noisier measurements are not over‐fitted. Suitable values are generally guided by reciprocal and stacking analyses (Binley and Slater 2020; Singha et al. 2022). Modern software packages such as ResIPy implement these procedures and enable systematic handling of acquisition parameters and data‐quality metrics (Blanchy et al. 2020; Singha et al. 2022). For expanded methodological details, including equations, array schematics, and photographic examples, readers are referred to Singha et al. (2022).

In the Yucatán Peninsula, ERT has been extensively applied to characterize the regional karst aquifer, including studies of the Ring of Cenotes, hydrological connectivity among cenotes, and variations in freshwater‐lens thickness or dissolution features near the coast (Gómez‐Nicolás et al. 2018; Andrade‐Gómez et al. 2019; Zamora‐Luria et al. 2020; Perera‐Burgos et al. 2024). Although these investigations demonstrate the versatility of ERT in inland and coastal‐adjacent settings, no peer‐reviewed marine ERT applications have been reported for the Yucatán region to date.

### Survey Design and Site Selection

Four ERT transects were positioned to contrast areas where the coastal aquitard is an intact and unfractured horizon on land with zones where it is breached by PSGD (Figures 1 and 2). *Transect A (Sisal)* placed perpendicular to the shoreline 100 m from monitoring well P7a, this profile crosses a sector where no structural discontinuities have been mapped. It provides a baseline resistivity–lithology calibration for an intact coastal aquitard and can be compared with geohydrological data previously reported by Canul‐Macario et al. (2020). *Transect B (Carbonera)* is located 16 km eastward from transect A in the Carbonera Lagoon, this line intersects petén Dzul‐Ha PSGD that rises through a fracture‐controlled perforation of the coastal aquitard beneath a mangrove swamp. The site offers a well‐documented inland PSGD where the resistivity response of a breached coastal aquitard can be imaged. *Transects C and D (Dzilam de Bravo)* are marine ERT seabed profiles surrounding the offshore PSGD Xbuya‐Ha. Transect C targets the presumed continuation of an intact coastal aquitard beneath shallow sea floor, whereas Transect D crosses the main submarine vent to capture the electrical signature of a fully breached horizon.

This A to D progression—from intact confinement to progressive fracturing on land, and finally to submarine breaching—allows the electrical model of the coastal aquitard to be “calibrated” in a controlled onshore environment and then tested where hydraulic conditions and structural setting are independently constrained by previous studies. Leveraging these well‐studied PSGD systems maximizes interpretive confidence while demonstrating the added value of a multi‐electrode ERT system in coastal‐karst settings where the method has so far been under‐utilized.

The study transects are situated within ecologically and environmentally valuable areas, all of which are under official protection due to their high biological significance. Transect A is located within the El Palmar Reserve; Transect B lies within the State Reserve of Marshes and Mangroves of the Northern Coast of Yucatán (Site 2468); and Transects C and D are situated within the Dzilam State Reserve, a wetland recognized under the Ramsar Convention for its international importance (Ramsar Convention Secretariat 2016).

### Field Data Acquisition, Inversion and Forward Modeling

ERT field surveys were performed with a SuperSting R8 multichannel system (Advanced Geosciences Inc. 2014) in a dipole–dipole configuration with 5 m electrode spacing (6 m for Transect A). Transect A, comprises 56 electrodes over 330 m. Transect B extends 120 m (25 electrodes) through the mangrove patch surrounding petén Dzul‐Ha along a NW–SE azimuth; dense vegetation limited array length and required setting electrodes 13‐14 within the spring pool at 60‐65 m from the start of the line. Marine Transects C and D, each 275 m long with 56 electrodes, were laid on the seabed surrounding the PSGD Xbuya‐Ha during neap low tide, when the water column averaged ~1.2 m. The procedure is fully described here, but a visual illustration of the installation can be seen in *Les Cénotes du Mexique* (Bonne Pioche 2020) for readers interested in additional context. Reciprocal measurements on C and D, stack‐error tests on B, and seasonal repeats of A provide quantitative estimates of acquisition noise and temporal variability (Gómez‐Nicolás et al. 2018).

Floating (towed) ERT arrays in ultra‐shallow settings rapidly lose sensitivity to sub‐seafloor resistive targets once water depth exceeds ~0.5‐1 m, whereas seabed electrode arrays remain sensitive for water depths of ~1‐5 m (Papadopoulos et al. 2022). Accordingly, given the ~1.2 m water depth during acquisition and the expected target depths, Transects C and D were collected with seabed‐installed electrodes rather than a towed array.

Raw data were screened to retain measurements with injected current >10 mA, voltage >0.1 mV, apparent resistivity between 0.01 and 100 Ω m, and transfer resistance (V/I) > 1 × 10^−4^ Ω, following manufacturer specifications (Advanced Geosciences Inc. 2014). Filtered datasets were inverted with ResIPy (Blanchy et al. 2020) using a smoothness‐constrained least‐squares algorithm. Error floors were iteratively adjusted until the reduced $\chi^{2}=\frac{1}{N}\sum_{i}{\left(\frac{d_{i}-f_{i}\left(m\right)}{\epsilon_{i}}\right)}^{2}$ approached unity, where $d_{i}$ are the observed data, $f_{i}\left(m\right)$ is the forward model for parameters m, $\epsilon_{i}$ are the data errors used for weighting, and N is the number of measurements.

Reciprocal and stacking error information was available across the four transects, but for Transects B and D we over‐rode reciprocal weighting and fixed the noise at *a_wgt* = 0, *b_wgt* = 0.01. The strong groundwater flow and mixing in these two lines create genuine forward–reverse differences; treating them as “error” (the reciprocal model yielded *b_wgt* = 0.022) suppressed the known springs at Xbuya‐Ha and the adjacent southern spring. A higher noise level would over‐smooth the inversion and mask these features because the apparent forward–reverse mismatch reflects transient fluid dynamics rather than structural variability. Using a 1% noise level therefore preserved the anomalies and more appropriately represents uncertainty in these hydrodynamically active sections.

**Figure 3:**
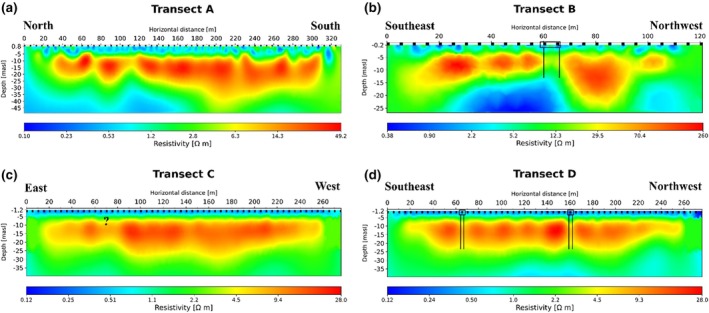
Inverted models of the field data (resistivity Ω·m) acquired along Transects A, B, C, and D (see Figures 1 and 2). Note that the minimum and maximum horizontal distances vary among the transects, and that the color scales for Transects C and D are identical, while Transects A and B each use their own distinct scale to better show features of the data. The black rectangles indicate the areas where PSGD are known to occur at the surface, while vertical lines mark inferred zones of aquitard breaching interpreted from the resistivity contrasts. Black dots along the x axis at the top of each section represent the electrodes. Interpretation toward the lateral and basal extents of the inverted sections is subject to increased uncertainty due to the progressive loss of sensitivity and data coverage inherent to ERT acquisition geometry.

After inversion and interpretation of the field data, we conducted a set of two‐dimensional forward‐modeling experiments to assess the offshore continuity of the coastal aquitard and the resistivity imprint of local breaches. All experiments share a common 2D model framework; the numerical domain, mesh, boundary conditions, and array geometry were held constant, while the properties and scenarios described below were varied. To parameterize the models, resistivity estimates for the main subsurface domains were obtained by applying statistical windows to the inverted sections (Figure 3), from which mean, minimum, maximum, and standard deviation values were extracted. In parallel, Archie's Law ($\rho_{r}=a\;\rho_{w}\;\phi^{-m}S_{w}^{-n}$, where a is the tortuosity factor, $\rho_{w}$ the resistivity of the saturating fluid, ϕ the effective porosity, m the cementation exponent, $S_{w}$ the water saturation, and n the saturation exponent) was used to compute apparent formation resistivities from pore‐fluid resistivities. Effective porosity of the coastal aquitard was independently obtained from x‐ray nano‐computed tomography analysis of a sample from well P7a (ϕ = 0.82%). Fluid resistivities were measured with a multiparametric sonde in monitoring wells P7a (Canul‐Macario et al. 2020) and P2 (Medina‐Rosado 2020) and Archie parameters (*a*, *m*, and *n*) were adopted from Perera‐Burgos et al. (2024). Inversion‐derived and Archie‐derived resistivities were compared to identify convergent values, which were adopted as representative inputs and iteratively refined by adjusting layer resistivities until synthetic responses reproduced the ERT field inverted sections.

PSGD resulting from breaches in the coastal aquitard were represented by vertical cylindrical anomalies 1 m‐wide and 5 m‐high (Vesilind and Skiles 2003). For these anomalies we explored resistivities spanning the range of groundwater and seawater conductivities (0.18 to 3.8 Ω·m) reported in the study area (Valle‐Levinson et al. 2011; Vera et al. 2012; Pacheco‐Castro et al. 2021). A two‐dimensional finite‐element mesh comprising about 45,000 triangular cells was generated in Gmsh (Geuzaine and Remacle 2009). Element sizes were refined from 0.5 m around the electrodes to 2 m at depth, to preserve aspect ratios suitable for potential‐field simulations. Zero‐normal‐current boundary conditions were imposed on the model flanks and base, which were positioned four array lengths from the electrodes to minimize edge effects. Synthetic data were produced in ResIPy (Blanchy et al. 2020) for a dipole–dipole array of 56 electrodes with *a* = 3 m and n_max = 8, mirroring the field configuration. Electrodes were assumed to be submerged, and their depths were varied around the field value of 1.2 m to examine the sensitivity of the responses to deployment depth. Gaussian noise at a 2% level was added to the data as uncorrelated, zero‐mean perturbations with a standard deviation equal to 2% of the measured apparent resistivity (Binley 2015).

The forward modeling experiments were designed as a hierarchical sensitivity analysis that progressively relaxes nearshore constraints and isolates the main controls on ERT detectability. The first set of experiments considers a nearshore PSGD, where vigorous freshwater outflow can measurably dilute the shallow marine layer and create a lateral surface‐salinity gradient. This scenario allows us to quantify how much conduit detectability depends on local seawater dilution, as opposed to subsurface structure alone. We developed two synthetic models. In the first model, a central zone with higher resistivity than the surrounding seawater (1.60 compared to 0.18 Ω·m) represents the contribution of freshwater from a hypothetical PSGD with a resistivity of 2.6 Ω·m, embedded within the coastal aquitard. The lateral extent of the zone influenced by the spring is based on the findings of Valle‐Levinson et al. (2011), who mapped the conductivity in the vicinity of the Xbuya‐Ha PSGD and estimated a radius of influence of approximately 40 m. Accordingly, this model depicts conditions characteristic of a highly active spring such as Xbuya‐Ha, with a discharge on the order of 1 m^3^ s^−1^. The second model is identical to the first, except that the marine layer is homogeneous, representing a scenario in which a conduit exists but discharge is insufficient to generate a brackish water body within the marine layer. These two models were used to quantify the effect of removing the surface salinity gradient on the ability of electrical resistivity tomography (ERT) to detect the conduit, specifically to determine the decline in the electrical signal and to evaluate the extent to which detection relies on local dilution of shallow marine waters.

Having bracketed the influence of a steady nearshore salinity gradient, we next incorporate tidal modulation because tides can substantially alter PSGD magnitude, as well as the geometry and intensity of the brackish plume. Accordingly, we conducted a series of experiments to simulate the tidal conditions reported by Valle‐Levinson et al. (2011) during low and high tides. For these models, the seawater column was divided into two horizontal layers. The upper layer represents seawater without PSGD influence and was assigned a uniform salinity equivalent to regional average seawater (0.18 Ω·m). The thickness of this layer, *h_*swsi (“sea without PSGD influence”), was treated as the independent parameter controlling tidal state and was systematically varied across model scenarios (Table 1).

**Table 1:** Thickness and Resistivity Parameters Used in the Forward Models Simulating Tidal Modulation of PSGD, Based on Tidal Conditions Reported by Valle‐Levinson et al. (2011) at Xbuya‐Ha. The Parameter h_swsi Denotes the Thickness of the Seawater Layer Without PSGD Influence

Case	h_swsi (m)	Width of the PSGD‐Influenced Surface Zone (m)	Resistivity of the PSGD‐Influenced Surface Zone (Ω m)	Resistivity of the PSGD Conduit (Ω m)
1	0	40	1.6	2.6
2	0.75	11	0.8	1.3
3	1.5	3	0.4	0.65
4	2.25	0.83	0.2	0.325
5	3	0.23	0.18	0.2

The lower layer corresponds to the PSGD‐influenced surface zone, defined as the portion of the shallow marine layer affected by freshwater discharge and associated salinity dilution. Its thickness, *h_*ssi (“sea with spring influence”), was held constant at 1.2 m, while its lateral extent and resistivity were adjusted as functions of *h_*swsi, following the relationships reported by Valle‐Levinson et al. (2011). Specifically, increasing *h_*swsi leads to a reduction in the width of the PSGD‐influenced surface zone by a factor of approximately 3.6 and to a halving of conductivity within both the surface dilution zone and the discharge conduit. Models were developed for each of these tidal scenarios to evaluate the response of ERT. The full set of thickness, width, and resistivity parameters employed in the modeling is presented in Table 1.

Once the influence of nearshore salinity structure, with and without tides, is bracketed, we shift to the key structural uncertainty relevant to mapping confinement, namely whether the coastal aquitard is present at all. To avoid conflating this question with changes in water depth, we first compare transects with and without the aquitard at a fixed shallow water depth representative of nearshore survey conditions. To this end, two models were constructed with and without the aquitard—excluding any karst conduits. In both cases, the model assumes a water depth of 1.2 m, as is characteristic of the area near Xbuya‐Ha.

Finally, we extend the same framework offshore, where increasing water depth is expected to dominate signal attenuation. This last set of experiments quantifies the practical limit at which an aquitard, if it extends seaward, becomes indistinguishable in seafloor ERT data as the overlying seawater column thickens. Prior work, particularly by Perry et al. (1989), suggests that the confining aquitard could extend up to 30 km offshore, albeit based on testimonies and qualitative observations. As water depth increases at an average rate of ~1 m / km offshore (Figure 1b), the question arises as to the limit at which the aquitard becomes undetectable by ERT. To address this, the previous experiment was rerun with water depth increments of 6 m up to a maximum of 30 m. It is important to note that the electrodes remain positioned on the seafloor; therefore, the origin of the z‐axis corresponds to the depth at which the electrode array is located.

## Results

### ERT Results along Transects A‐D

#### Transect A (330 m; Coastal Dunes near Monitoring Well P7a)

The inland section that extends ~300 m from the coast and passes within ~50 m of monitoring well P7a and across monitoring well P2, images three electrically distinct zones in the upper ~30 m (Figure 3a) that are consistent with the local lithostratigraphy. The upper 5 m shows low resistivity relative to the deeper parts of the profile (<~1 Ω m), compatible with Holocene coastal dune deposits of sandy limestone with shell fragments. Beneath this, a relatively resistive, laterally persistent horizon (typically ~14‐50 Ω m) occurs at ~−9 to −20 masl and is interpreted as the dense, recrystallized interval intercepted by well P7a that acts as the coastal aquitard. Below ~20 m, resistivity drops sharply to values of a few Ω m and remains low to the maximum depth imaged (50 m), consistent with saline–brackish saturation of karstified Miocene–Pliocene limestone forming the regional aquifer.

Two apparent anomalies on the left‐hand side of Transect A are expressed as ~1‐2 m undulations in the top of the resistive horizon (at approximately x 70 m and x 125 m); they coincide with near‐surface heterogeneity associated with unvegetated dune sands and adjacent vegetated crests, as documented by drone imagery provided in the Supporting Information. These surface conditions produced strongly spatially variable electrode contact resistance along Transect A, with values locally reaching ~5000 Ω, whereas contact resistance along Transects B‐D remained comparatively low (typically <~200 Ω). Within the resolution of this line, we therefore interpret the aquitard as laterally continuous, and we attribute the two apparent “anomalies” to near‐surface conditions rather than subsurface breaches.

#### Transect B (120 m across the Dzul‐Ha petén, La Carbonera Lagoon)

Figure 3b shows the 120‐m ERT line crossing the circular spring pool of Dzul‐Ha between *x* ~57‐65 m. The shallow subsurface (< −5 masl) is characterized by low resistivities, consistent with the local depositional setting, where thin lagoonal sediments replace the coastal dune sands. Beneath this shallow conductive layer, the inversion reveals a vertically elongated conductive zone around 60‐65 m, with resistivities on the order of ~6‐14 Ω m extending from the shallow subsurface to ~−20 to −27 masl. This feature is interpreted as a karst conduit breaching the coastal aquitard. On either flank of this feature (*x* ~15‐60 m and 70‐105 m), more resistive domains (>~30 Ω m) extend to ~−5 to −18 masl and are interpreted as the dense coastal aquitard, which here lies close to the surface owing to the absence of coastal dune sands.

The inverted resistivity structure suggests that the conduit extends vertically at least ~10 m beneath the spring pool, with an entrance diameter of a few meters. The greater apparent width of the feature in the resistivity model likely reflects inversion smoothing and geometric regularization that limit resolution of narrow structures. While the deeper geometry and connectivity of the conduit system remain unresolved, the conductive anomaly beneath the pool is robust across inversion settings despite local uncertainty introduced by electrode non‐collinearity and standing water in the mangrove. These results thus clearly indicate a focused vertical pathway for lagoon–aquifer exchange consistent with documented tidal reversals at Dzul‐Ha.

#### Transect C (275 m; Seabed Line Proximal to Xbuya‐Ha)

Acquired at neap low tide (mean water depth ~1.2 m) with the water layer left unconstrained in the inversion, this marine section (Figure 3c) images a 5 m thick low resistivity band at shallow depth with only subtle lateral variations. Beneath this a broad, laterally continuous band of moderate resistivity (~10 to ~28 Ω m) extends downward from −8 to −20 masl, progressively transitioning to a uniformly conductive domain at depths greater than ~28‐30 m. The lack of lateral variability along this line, which runs parallel to the coast, is consistent with the observation that the line crosses no known vents.

Shallow stratigraphic contacts anticipated from regional geology (e.g., Holocene marine sediments–coastal aquitard near ~5 m; Pleistocene–Neogene near ~10 m) are not continuously expressed, which we attribute to true heterogeneity in rock properties and marine acquisition effects (water‐column variability and lateral conductivity gradients in conductivity of the water column). The E–W orientation places this line transverse to the expected inland‐to‐offshore conduit trajectories; the continuous mid‐depth “freshened band” (*x* ~60‐80 m) may reflect an oblique intersection with the broader conduit zone with no associated discrete vents (Vesilind and Skiles 2003).

#### Transect D (275 m; Seabed across the Xbuya‐Ha PSGD)

Also collected at neap low tide with an unconstrained water layer, this section (Figure 3d) shows a predominantly conductive marine matrix with localized lower‐resistivity zones (annotated by black lines), located between approximately −10 and −25 masl near *x* ~65 m and *x* ~160 m. Their amplitudes (peaking, ~30 Ω m) and positions coincide with a secondary spring and the main Xbuya‐Ha vent, respectively. Below ~25 m, the subsurface appears more homogeneous and conductive than the overlying layer, suggesting a transition to a deeper, uniform conductive domain.

The two shallow contacts inferred from regional stratigraphy (that along the coast occur at a depth of ~−5 masl and ~−10 masl) are not resolved as continuous horizons in this marine setting. This may be due to spatial variation in pore water salinity and strong lateral conductivity gradients, as seen most clearly in localized freshened pathways near the vents. The geometry supports discrete freshwater conduits embedded in a saline matrix and is consistent with the known tidally reversing flow regime at Xbuya‐Ha.

### Forward Modeling the ERT Signature of an Offshore Aquitard

#### Conceptual Model of Electrical Units Based on ERT Data and Monitoring Wells Logs

Across Transects A‐D, the inverted ERT sections consistently resolve a sequence of subhorizontal layers (Figure 3). For the marine Transects (C and D), the uppermost layer (U0) corresponds to a marine unit with resistivities ranging from 0.18 to 1.6 Ω·m. This is underlain by an intermediate unit (U1) with bulk resistivities of 1‐2 Ω·m and a markedly resistive (~250 Ω·m) horizon (U2) that we interpret to represent the coastal aquitard. Beneath this horizon, a more conductive (2 Ω·m) basal domain (U3) is observed, consistent with the high‐porosity limestone of the CPF. These domains define the framework adopted for subsequent forward modeling.

Archie‐derived formation resistivities were computed using a tortuosity factor a=1 and cementation exponent m=1.95 (Perera‐Burgos et al. 2024), porosities of 40% for marine sediments and 30% for the CPF, full saturation (S=1), and a pore‐fluid resistivity of 0.2 Ω·m (approximating seawater). The resulting values closely match those obtained from the inverted sections, providing independent support for the representative resistivities assigned to each domain.

The resistivity of the dissolution conduits (100% porosity) falls within the range expected from pore‐water conductivities documented in the region—2.6 Ω·m for the freshest groundwater (3.8 mS cm^−1^; ~7% seawater), 1.6 Ω·m for more brackish lens water (6.3 mS cm^−1^; ~11% seawater), and 0.264 Ω·m for saline‐dominated discharge (37.9 mS cm^−1^; ~68% seawater) (Valle‐Levinson et al. 2011; Vera et al. 2012; Pacheco‐Castro et al. 2021). These values were used to simulate the presence of localized breaches in the coastal aquitard (0.08% porosity) capable of allowing upward flow of confined groundwater.

#### Influence of a Surface Salinity Gradient on Anomaly Detection

**Figure 4:**
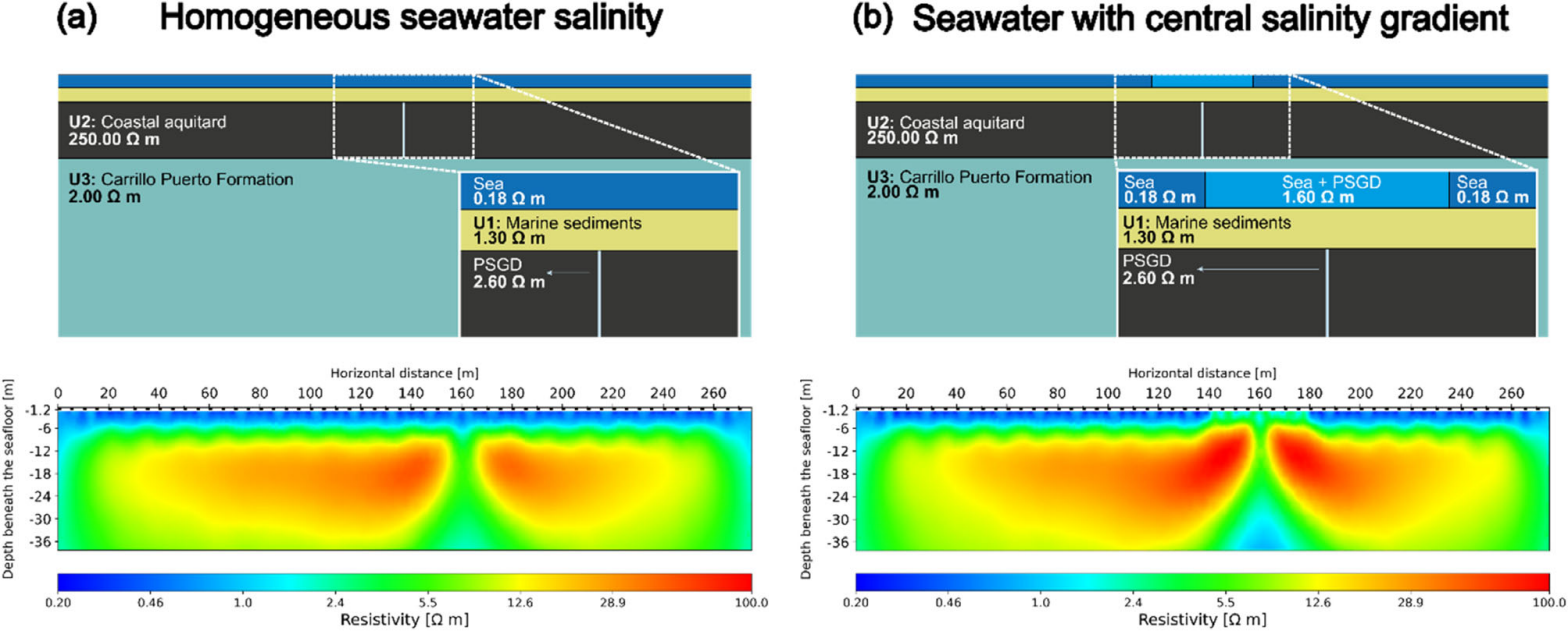
Results of the forward modeling with (a) a seawater layer of homogeneous salinity compared with (b) a seawater layer exhibiting a central salinity gradient.

The two models, with and without local dilution of the shallow coastal ocean (Figure 4) show that the presence of a PSGD‐induced salinity gradient substantially amplifies, but does not determine, anomaly detectability. With a relatively extensive area of dilution, as shown in Figure 4b, the inverted model exhibits a distinctive signature expressed as a pair of high‐resistivity bands (~100 Ω·m) centered over the portion of the sea affected by PSGD. In contrast, Figure 4a, with a homogeneous marine layer, produces a weaker contrast (~50 Ω·m). Nevertheless, the conduit remains morphologically visible at the expected location. From this experiment, we infer that conduits at this level of resistivity contrast remain detectable even in the absence of a pronounced surface salinity gradient, although the signal amplitude is reduced by approximately 50% compared to the scenario in which a PSGD‐induced surface salinity gradient is present (Figure 4b).

#### Electrical Resistivity Response to Tidal Modulation

**Figure 5:**
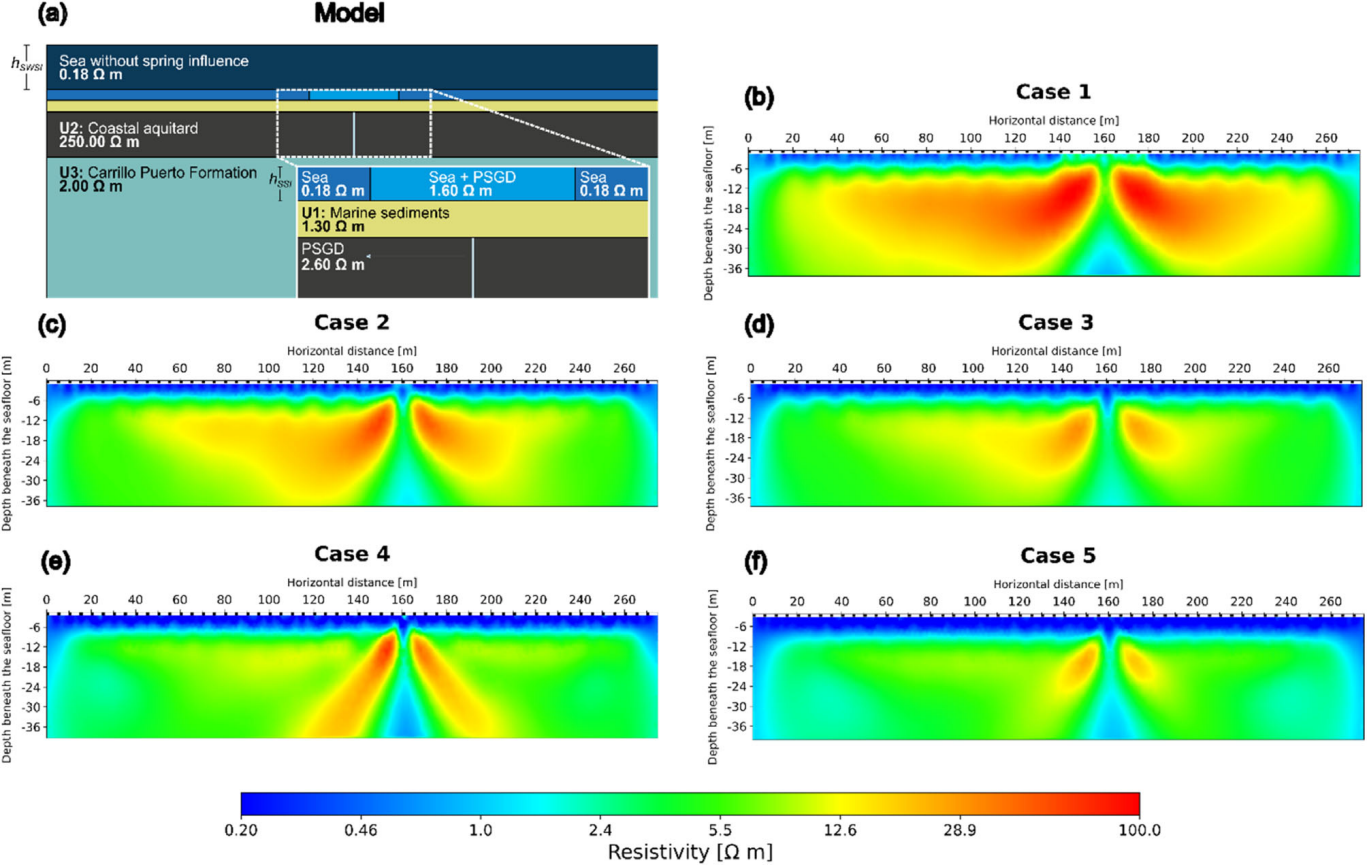
Electrical resistivity response to tidal modulation of PSGD. In the model the water column is represented by two layers: (i) a layer influenced by PSGD (h_ssi thickness) with a salinity gradient and a more resistive core of fresher water, and (ii) an upper layer of uniform salinity (h_swsi thickness) with a resistivity of 0.18 Ω·m. The tidal scenarios replicate the conditions reported by Valle‐Levinson et al. (2011) for the same area. An increase in h_swsi reduces the PSGD‐influenced zone by a factor of approximately 3.6 and decreases conductivity by half in both the PSGD zone and the discharge conduit (parameters for Case 1 to 5 are given in Table 1).

The results in Figure 5 show that, despite tidal variations, the anomaly associated with PSGD remains detectable. However, with changing parameter values from Case 1 to 5 (Table 1), there is a progressive loss in the precision of its delineation, both in terms of its lateral extent and its position along the x‐axis.

#### Distinguishing the Presence or Absence of the Coastal Aquitard

**Figure 6:**
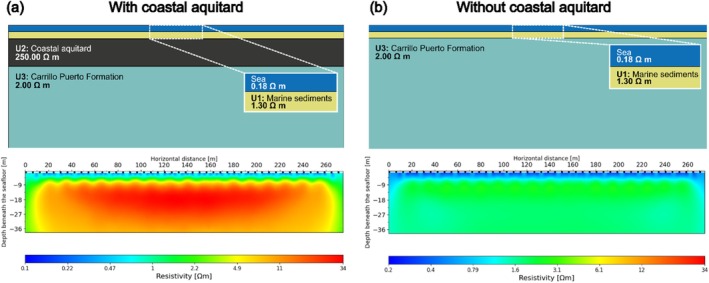
Electrical resistivity models (a) with and (b) without a coastal aquitard. When the aquitard is present, peak resistivity rises to ~33 Ω m and a localized resistive zone shows contrasts up to ~20 Ω m; without it, the response is nearly uniform with maxima of ~3 Ω m and variations <1 Ω m. Karst conduits are not included.

Using resistivity parameters for electrical units defined above, the presence of the aquitard has a significant (order of magnitude) effect on the electrical signal. In models that include the aquitard, the maximum resistivity reaches approximately 33 Ω m, compared with a maximum of approximately 3 Ω m with no offshore aquitard. With the aquitard (Figure 6a), a resistive zone is observed with contrasts nearing 20 Ω m, while without the aquitard (Figure 6b), almost the entire model exhibits values that do not exceed 1 Ω m. The morphological signature is not dramatically distinct; therefore, the detection of the aquitard strongly depends on prior knowledge of its expected signal and magnitude at a given water depth.

#### Depth‐Dependent Attenuation of the Marine ERT Signal

**Figure 7:**
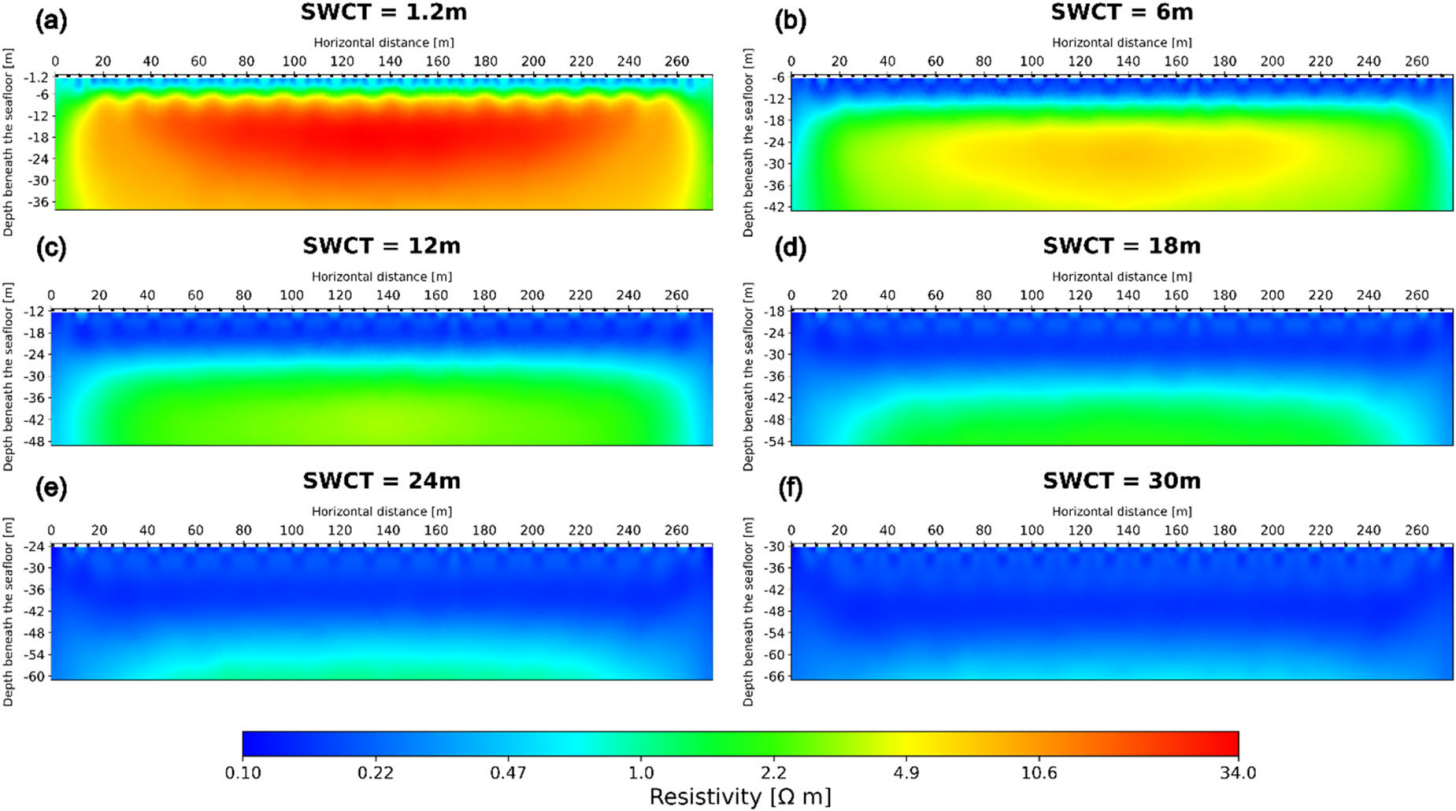
Attenuation of the marine ERT signal from an offshore aquitard with increasing seawater column thickness (SWCT). The panels show the forward‐modeled ERT response using the same layered geometry, thicknesses, and initial electrical resistivities defined in Figure 6a. Note the side z‐axis is depth beneath the sea surface. The maximum modeled resistivity decays exponentially with water depth: greater water depth progressively dampens the sub‐seafloor resistivity response and reduces array sensitivity.

In the analysis of the maximum resistivity values obtained from the marine ERT model that includes an aquitard, a systematic decay in the maximum resistivity response (*y* in Ω m) is observed as the water column depth (*x* in m) increases (Figure 7). This decay follows an exponential law of the form:

$$y\left(x\right)\approx 22.{\text{9e}}^{\left(-0.129x\right)}$$

The attenuation coefficient $\frac{1}{\lambda }$ = 0.129 implies a characteristic depth of approximately 7.8 m, indicating that for every additional 7.8 m of seawater above the electrodes, the maximum resistivity response is reduced to 37% of its previous value. It is important to note that this decay relationship and the derived characteristic depth are strictly valid only for the specific model configuration employed in this study, including the prescribed aquitard thickness, layer geometry, and electrical resistivity contrasts defined in the Methods section. This behavior reflects the electrical attenuation induced by the increasing water column, which diminishes the array's sensitivity to the solid medium beneath the seafloor. A similar depth‐dependent loss of sensitivity has been documented in marine ERT of tidally modulated freshwater discharge, where increased tidal stage focuses current within the conductive seawater layer and the inversion progressively fails to recover the underlying resistive freshwater body (Henderson et al. 2010). Thus, greater water thickness predictably weakens the subsurface resistivity signal, following an exponential decay pattern typical of energy loss and dispersion in conductive media.

## Discussion

This study evaluates the hydrogeological conditions under which marine ERT can reliably detect and interpret offshore aquitards and point‐source submarine groundwater discharge (PSGD) in a coastal karst system. Our objectives were to determine the limits of aquitard detectability as a function of electrical contrast, thickness, and water depth; to assess the sensitivity of marine ERT to vertical pathways breaching the coastal aquitard; and to examine how attenuation of water‐column salinity structure affects anomaly detection.

Field observations and forward modeling show that marine ERT performs robustly only under a restricted set of conditions. Detectability is highest where PSGD fluxes are large and generate strong freshwater–seawater contrasts, as at Xbuya‐Ha. Clear imaging of vertical conduits requires a well‐defined, resistive aquitard that contrasts sharply with surrounding saline‐saturated units. Finally, the method is effective only in very shallow water, as even modest increases in water‐column thickness cause rapid signal attenuation and loss of resolution.

The constrained performance of marine ERT identified here should be interpreted as a defining characteristic of the method rather than a shortcoming. By explicitly delineating the hydrogeological conditions under which marine ERT is effective, this study provides practical guidance on the circumstances under which the technique can contribute meaningful information and when alternative approaches are more appropriate. The primary contribution is therefore not the demonstration of universal detectability of PSGD, but the establishment of realistic performance bounds under field conditions.

A key advantage of marine ERT is its non‐invasive nature. Many PSGD systems (including those studied here) occur in ecologically protected coastal lagoons, mangroves, or shallow marine environments where drilling, coring, or permanent installations are restricted, costly, or risk perturbing the natural hydraulic regime and ecology. In such settings, marine ERT enables subsurface characterization while preserving natural confinement, a critical requirement in environmentally sensitive coastal systems.

Importantly, the value of marine ERT extends beyond the direct detection of active discharge conduits. Even where electrical contrasts are insufficient to resolve PSGD unequivocally, resistivity sections provide first‐order constraints on stratigraphic architecture, aquitard continuity, and the distribution of conductive and resistive domains. These constraints refine conceptual models of coastal aquifers and inform interpretations of groundwater–seawater interaction, even in the absence of a clear PSGD anomaly (e.g., Islas‐Domínguez 2020).

Although Xbuya‐Ha represents an unusually vigorous example of PSGD, focused coastal and submarine springs are documented worldwide. While such features are commonly associated with karstic carbonate settings (Murgulet et al. 2020), analogous focused discharge pathways have also been identified in volcanic terrains through lava tubes (Attias et al. 2020). Recent global inventories further indicate that submarine springs are widespread and not restricted to isolated or anomalous environments (Bouimouass et al. 2024).

The strong dependence of detectability on freshwater–seawater electrical contrast suggests that marine ERT is best applied as a targeted tool, deployed during hydrodynamic conditions that enhance contrast, such as low tide or periods following intense recharge. Rather than limiting applicability, this sensitivity highlights a niche role for ERT as an event‐based or time‐constrained method capable of capturing transient hydrological states.

Marine ERT is therefore most appropriately viewed as a screening and decision‐support tool within a broader, multidisciplinary framework. Positive anomalies can guide the placement of focused hydrogeochemical or hydrodynamic measurements, while weak or null responses constrain aquitard continuity and the offshore extent of freshened groundwater. In this context, negative results can be informative and reduce uncertainty rather than indicating methodological failure.

Our experiments demonstrate that seabed‐installed marine ERT can detect dissolution conduits associated with PSGD as vigorous as that of Xbuya‐Ha. However, this spring represents a special case within the karst system: its discharge, on the order of 0.5 m^3^ s^−1^, is exceptionally high and uncommon according to the available literature. Xbuya‐Ha was selected because it is the most thoroughly documented spring in the region and the only site with robust reference data for comparison; nonetheless, this choice likely over‐represents favorable conditions and should be interpreted as defining an upper bound on detectability. In more common contexts, lower discharge rates, less resistive aquitards, or thicker water columns, target bodies will appear fainter, more diffuse geometries, and vertical resolution will decrease. For regional surveys, the towed mode can serve as a screening tool for shallow anomalies, but delineating the base of the aquitard and the extent of the interface requires lines with fixed electrodes, denser sampling, and multiparameter integration (Henderson et al. 2010; Cantarero et al. 2019; Costall et al. 2020). In summary, our results for Xbuya‐Ha should be regarded as an upper bound on performance for detecting dissolution conduits linked to PSGD. Generalization to other sites requires adjusting the electrical contrasts, accounting for the water column effect in array design, and, above all, triangulating interpretation with hydrogeological and solute transport evidence.

The forward models assume a relatively resistive aquitard (~250 Ω·m), consistent with diagenetically cemented horizons such as marine hard grounds and calcretes formed under arid conditions in carbonate and non‐carbonate terranes (Santos et al. 2021; Vallack 2021). This assumption enhances contrast with seawater‐saturated porous units and facilitates visualization of PSGD conduits. However, it represents an optimistic scenario: if the aquitard exhibits lower resistivity (e.g., due to disseminated clays or filled fractures), the contrast will decrease, and the target bodies will attenuate or merge with the background.

The models presented here rely on idealized geometries and simplifying assumptions, including sharp geoelectrical interfaces, local homogeneity, quasi‐steady conditions, and characteristic conduit resistivities. Given the equifinality of the problem, we recommend that marine ERT studies incorporate sensitivity analyses of aquitard resistivity and salinity, resolution tests and depth‐of‐investigation estimates; interpretation in conjunction with hydrogeochemical tracers (e.g., radon, radium, temperature) and cross‐validation using independent hydrogeological and hydrodynamic data (Henderson et al. 2010; Cantarero et al. 2019; Costall et al. 2020).

Through open availability of the inversion workflows, forward models, and processing scripts openly available (Gómez Nicolás et al. 2025), this study is intended to serve as a reproducible methodological reference rather than a site‐specific demonstration. Open access to these materials allows readers to test alternative assumptions regarding aquitard properties, pore‐fluid salinity, and conduit geometry, and to evaluate how detectability thresholds shift under different hydrogeological conditions. As new petrophysical or borehole data become available, the framework can be readily adapted to refine performance bounds and support optimized survey design and integrated interpretation in other coastal settings.

## Conclusions

This study demonstrates that marine ERT can be an effective, non‐invasive approach for imaging PSGD and the offshore stratigraphic controls that govern it, when deployed under favorable hydrogeological and geometric conditions. Through the first marine ERT characterization of PSGD in the Yucatán Peninsula, we show that vertical dissolution conduits can be resolved with high confidence where vigorous discharge produces strong electrical contrasts across a laterally continuous, resistive coastal aquitard in shallow water.

The principal contribution of this work is the definition of practical performance limits for geoelectrical imaging in karstic marine environments. Forward modeling demonstrates that ERT sensitivity decays exponentially with water‐column thickness, with a characteristic depth of approximately 8 m, such that the resistivity response is reduced to ~37% for each additional 8 m of water above the seabed electrodes. This quantitative benchmark provides a useful constraint for survey planning, indicating that seabed‐installed arrays offer superior resolution of shallow sub‐seafloor targets, but that their effectiveness is rapidly diminished by increasing water depth and saline attenuation.

Beyond site‐specific characterization of the Xbuya‐Ha and Dzul‐Ha systems, this study establishes a transferable methodological framework that integrates field observations, forward modeling, and Archie‐based petrophysical relationships to evaluate PSGD detectability prior to data acquisition. Rather than offering a universal solution, our results define an upper bound on marine ERT performance in coastal karst settings and highlight the need to pair resistivity imaging with independent hydrogeological and geochemical observations to reliably interpret flow pathways and discharge processes.

## Authors' Note

The authors do not have any conflicts of interest or financial disclosures to report.

## Supporting information


**Figure S1.** A priori information is used to constrain the geoelectrical model. Lithostratigraphic column and electrical conductivity measurements from boreholes P7a (located on the coastal dune, ~300 m from the shoreline) and P2 (~65 m from the shoreline). Modified from Canul‐Macario et al. (2020).
**Figure S2.** Comparison between field‐measured electrode contact resistance and the inverted resistivity model along Transect A at Sisal. The upper panel shows contact resistance values for individual electrodes plotted along horizontal distance, with reference thresholds recommended by the instrument manufacturer (AGI, Advanced Geosciences Inc.). The lower panel shows the corresponding inverted resistivity section. Horizontal distance of 0 m corresponds to the shoreline, whereas 320 m marks the most landward extent of the transect. Zones of elevated contact resistance coincide with shallow, resistive surface conditions imaged in the inversion.
**Figure S3.** Aerial drone photograph of the Sisal study area showing the location of the electrical resistivity tomography profile (Transect A) and its proximity to the monitoring wells P2 and P7a. The yellow line marks the ERT transect, and red squares indicate monitoring well locations. Areas of sparse vegetation visible along the transect coincide with zones of elevated electrode contact resistance.
**Figure S4.** Evolution of the root mean square error (RMSE) during the inversion process for the four electrical resistivity tomography (ERT) transects (A–D). In all cases, the inversion was iterated until a target misfit of less than 1.5% was achieved, indicating stable convergence and an acceptable fit between observed and modeled data.

## Data Availability

The raw ERT measurements, inversion models, and processing scripts are openly available in Zenodo at https://doi.org/10.5281/zenodo.16879253 under the CC‐BY 4.0 license.
