# Supplementary material for: Imaging Point Source Groundwater Discharges in a Confined Coastal Aquifer Using Electrical Resistivity
Source: Ground Water. 2026 Apr 27;64(4):405–21. doi: 10.1111/gwat.70071 (PMC13353669; doi:10.1111/gwat.70071)
Supplement: Supplementary file 1 — Figure S1. A priori information is used to constrain the geoelectrical model. Lithostratigraphic column and electrical conductivity measurements from boreholes P7a (located on the coastal dune, ~300 m from the shoreline) and P2 (~65 m from the shoreline). Modified from Canul‐Macario et al. (2020). Figure S2. Comparison between field‐measured electrode contact resistance and the inverted resistivity model along Transect A at Sisal. The upper panel shows contact resistance values for individual electrodes plotted along horizontal distance, with reference thresholds recommended by the instrument manufacturer (AGI, Advanced Geosciences Inc.). The lower panel shows the corresponding inverted resistivity section. Horizontal distance of 0 m corresponds to the shoreline, whereas 320 m marks the most landward extent of the transect. Zones of elevated contact resistance coincide with shallow, resistive surface conditions imaged in the inversion. Figure S3. Aerial drone photograph of the Sisal study area showing the location of the electrical resistivity tomography profile (Transect A) and its proximity to the monitoring wells P2 and P7a. The yellow line marks the ERT transect, and red squares indicate monitoring well locations. Areas of sparse vegetation visible along the transect coincide with zones of elevated electrode contact resistance. Figure S4. Evolution of the root mean square error (RMSE) during the inversion process for the four electrical resistivity tomography (ERT) transects (A–D). In all cases, the inversion was iterated until a target misfit of less than 1.5% was achieved, indicating stable convergence and an acceptable fit between observed and modeled data. [file GWAT-64-405-s001.docx]

Supporting Information


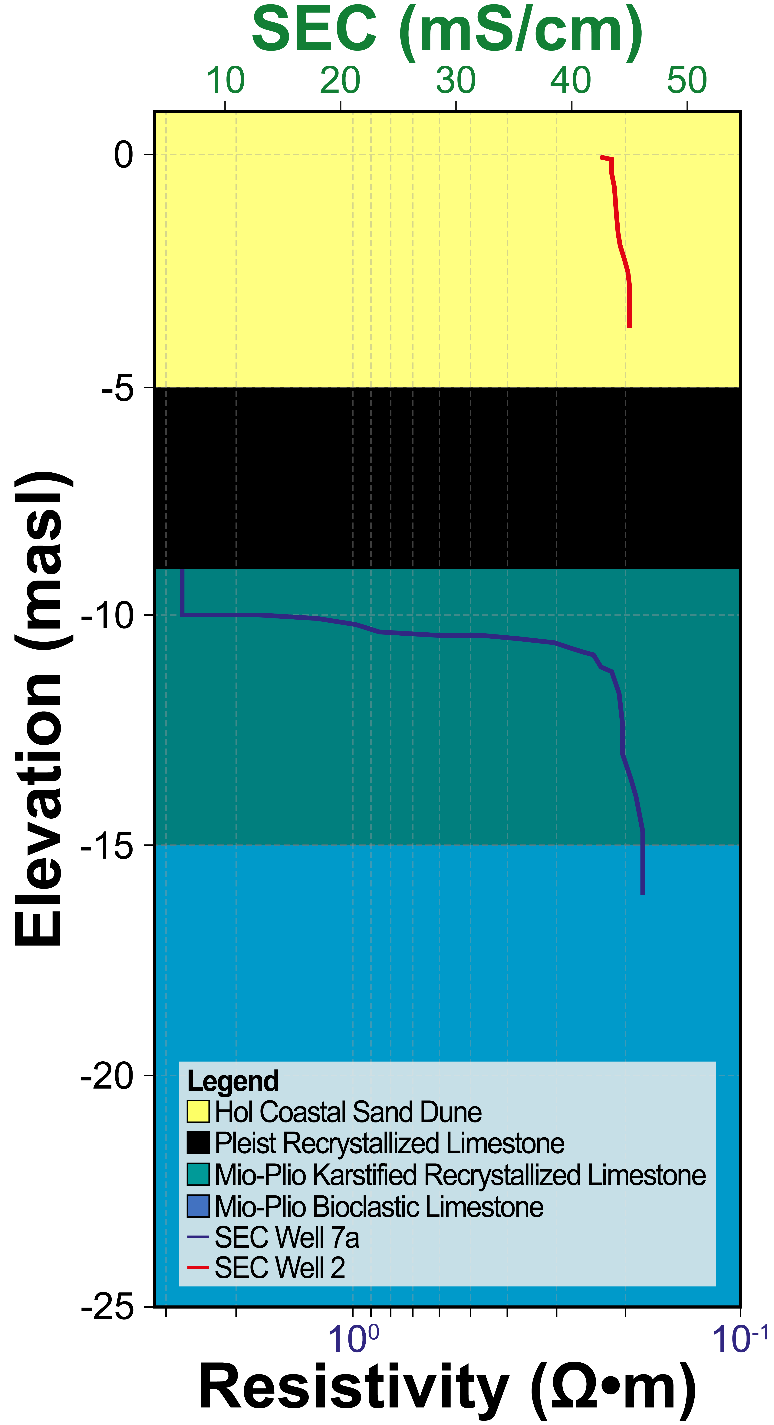


#### [Figure](#figur_errors) S1. A priori information is used to constrain the geoelectrical model. Lithostratigraphic column and electrical conductivity measurements from boreholes P7a (located on the coastal dune, ~300 m from the shoreline) and P2 (~65 m from the shoreline). Modified from Canul-Macario et al., (2020).


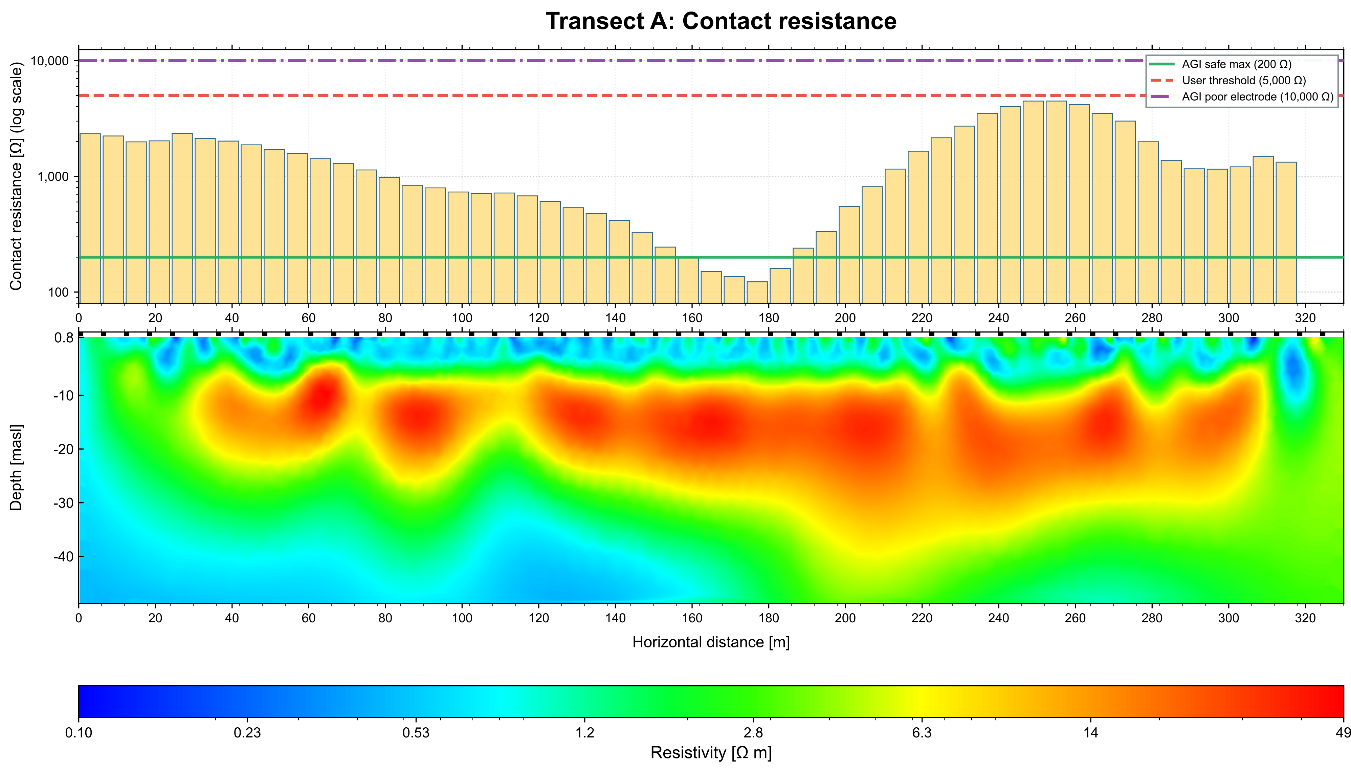


Figure S2. Comparison between field-measured electrode contact resistance and the inverted resistivity model along Transect A at Sisal. The upper panel shows contact resistance values for individual electrodes plotted along horizontal distance, with reference thresholds recommended by the instrument manufacturer (AGI, Advanced Geosciences Inc.). The lower panel shows the corresponding inverted resistivity section. Horizontal distance of 0 m corresponds to the shoreline, whereas 320 m marks the most landward extent of the transect. Zones of elevated contact resistance coincide with shallow, resistive surface conditions imaged in the inversion.


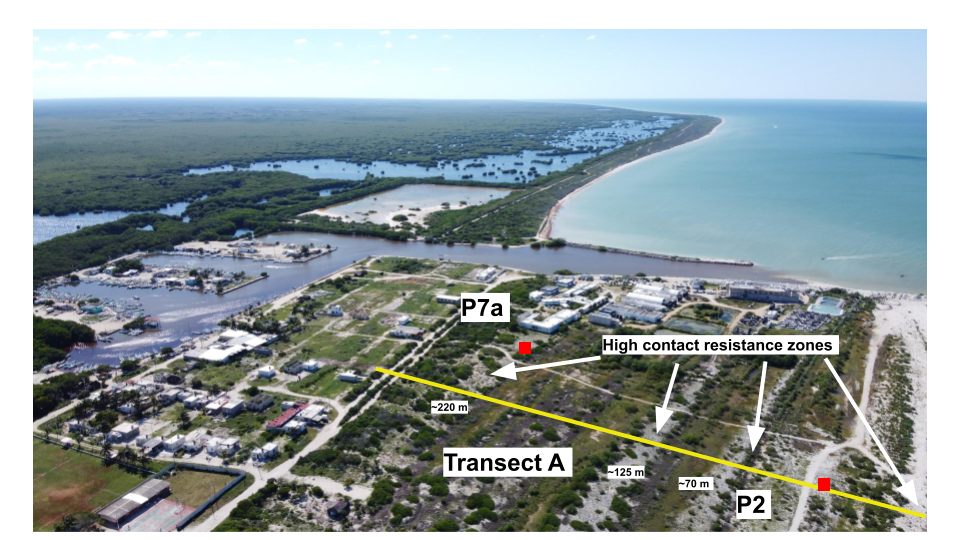


Figure S3. Aerial drone photograph of the Sisal study area showing the location of the electrical resistivity tomography profile (Transect A) and its proximity to the monitoring wells P2 and P7a. The yellow line marks the ERT transect, and red squares indicate monitoring well locations. Areas of sparse vegetation visible along the transect coincide with zones of elevated electrode contact resistance.


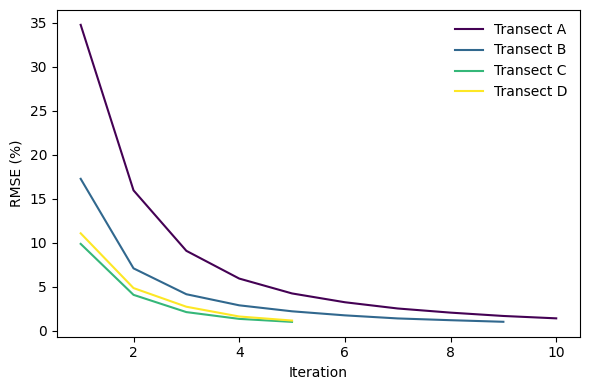


Figure S4. Evolution of the root mean square error (RMSE) during the inversion process for the four electrical resistivity tomography (ERT) transects (A–D). In all cases, the inversion was iterated until a target misfit of less than 1.5% was achieved, indicating stable convergence and an acceptable fit between observed and modeled data.
